# Integrated analysis of RNA-binding proteins in thyroid cancer

**DOI:** 10.1371/journal.pone.0247836

**Published:** 2021-03-12

**Authors:** Jing Zhen, Zhe Song, WenJie Su, Qing-Cui Zeng, JiaCen Li, Qin Sun

**Affiliations:** 1 Department of Geriatric Endocrinology, Sichuan Academy of Medical Sciences and Sichuan Provincial People’s Hospital, Chengdu, P.R. China; 2 Medical Center of Vascular Surgery and Thyroid, Sichuan Academy of Medical Sciences and Sichuan Provincial People’s Hospital, Chengdu, P.R. China; 3 Department of Anesthesiology, Sichuan Academy of Medical Sciences and Sichuan Provincial People’s Hospital, Chengdu, P.R. China; 4 Department of Geriatric Intensive Care Unit, Sichuan Academy of Medical Sciences and Sichuan Provincial People’s Hospital, Chengdu, P.R. China; University of Ulsan College of Medicine, REPUBLIC OF KOREA

## Abstract

Recently, the incidence of thyroid cancer (THCA) has been on the rise. RNA binding proteins (RBPs) and their abnormal expression are closely related to the emergence and pathogenesis of tumor diseases. In this study, we obtained gene expression data and corresponding clinical information from the TCGA database. A total of 162 aberrantly expressed RBPs were obtained, comprising 92 up-regulated and 70 down-regulated RBPs. Then, we performed a functional enrichment analysis and constructed a PPI network. Through univariate Cox regression analysis of key genes and found that *NOLC1* (*p =* 0.036), *RPS27L* (*p =* 0.011), *TDRD9* (*p =* 0.016), *TDRD6* (*p =* 0.002), *IFIT2* (*p =* 0.037), and *IFIT3* (*p =* 0.02) were significantly related to the prognosis. Through the online website Kaplan-Meier plotter and multivariate Cox analysis, we identified 2 RBP-coding genes (*RPS27L* and *IFIT3*) to construct a predictive model in the entire TCGA dataset and then validate in two subsets. In-depth analysis revealed that the data gave by this model, the patient’s high-risk score is very closely related to the overall survival rate difference (*p* = 0.038). Further, we investigated the correlation between the model and the clinic, and the results indicated that the high-risk was in the male group (*p* = 0.011) and the T3-4 group (*p* = 0.046) was associated with a poor prognosis. On the whole, the conclusions of our research this time can make it possible to find more insights into the research on the pathogenesis of THCA, this could be beneficial for individualized treatment and medical decision making.

## 1. Introduction

The thyroid gland is an endocrine organ located in the neck of human body and secretes two hormones thyroxine and triiodothyronine that are essential for the normal functioning of all the cells as well as regulating human metabolism, controlling weight, blood pressure, heart rate, and body temperature [1–4]. Thyroid cancer arises from the parafollicular or follicular cells and considered as one of the most common malignant tumor of endocrine system. Over the past 30 years, the incidence of thyroid cancer has been on the rise, each year it comprises for 3.4% of all tumor cases diagnosed globally [5–10]. The most famous types of thyroid tumors are papillary thyroid cancer (PTC) and follicular thyroid cancer with a better prognosis, and their survival rate can exceed 90% in the long-term development process. At present, PTC patients usually cured with conventional treatments including surgical treatment, radioactive iodine therapy or thyroid stimulating hormone suppression therapy [11–13]. Although most PTCs remain inert and survival rate of the patients is maximum, the recurrence and metastasis of tumors hinder the clinical treatment and resulted in poor prognosis [14–20]. For patients with locally advanced or distant metastatic PTC, the existing treatment methods are not enough and there is a need for a novel approach to diagnose and treat PTC.

RNA-binding proteins (RBPs) can interact with many types of RNA and have a very high appearance rate in various cells [21–25]. A total of 1542 RBPs were identified in human cells through high-throughput screening, accounting for 7.5% of all protein-coding genes [26–30]. RBPs refers to a kind of pleiotropic protein. RBPs transcribed after interacting with the target RNA, and finally regulate the level of gene expression. Previous pieces of research work revealed that RNA-binding proteins are involved in RNA metabolism and play a vital role in regulating RNA stability, alternative splicing, modification, localization, and translation [31–34]. Therefore, abnormalities in the expression of RBPs can cause several disorders including muscle atrophy, metabolic disorders, cancer, and germ cell development [35–41]. Dysregulated RBPs have been reported to mediate cell proliferation, apoptosis, angiogenesis, senescence, and metastasis in various cancer cells, the critical biological mechanisms of which include miRNAs regulation, alternative splicing, alternative Polyadenylation, RNA localization, RNA stability, and translational regulation [42–44]. Besides, some of these RBPs dysregulations are significantly associated with the prognosis of specific cancer patients. On the other hand, based on RBPs’ functional role in cancer, several therapeutics targeting RBPs including small-molecule drugs, inhibitors, small peptides, and antisense oligonucleotides have been developed and applied in clinical trials [45–47]. However, till now RBPs have not been well explored in thyroid cancer. In the emergence and development of THCA, the role RBPs is still unclear. Although the treatment of THCA has achieved great results in the last ten years, due to the high incidence of tumor-specific deaths, it is still worth considering the prognosis of patients and the need for new treatment methods. Thus, the analysis of RBPs in THCA can provide new insights as potential biomarkers for treatment and pathogenesis of thyroid tumors.

In the current investigation, we collected the THCA data from the TCGA database and conducted various experiments to assess the potential molecular functions and clinical value of RBPs in THCA. In addition, we also analyzed many differentially expressed RBPs that were closely related to THCA. Meanwhile, we carried out enrichment analysis on the GO and KEGG pathways, in order to demonstrate the possible mechanism of RBP in THCA. We also established a model related to the prognosis and tested the accuracy of the model through survival analysis, ROC analysis, univariate Cox analysis, and multivariate Cox analysis.

## 2. Materials and methods

### 2.1 Datasets

We downloaded the THCA transcriptome expression profile and corresponding clinical information from the TCGA database (https://portal.gdc.cancer.gov/repository). The expression data was HTSeq-FPKM type, containing 510 THCA tissues and 58 adjacent nontumorous tissue samples.

### 2.2 Identification of DEGs and functional annotations

We collected 1542 genes related to RBP from the literature [21]. Then we extract RBPs-related genes from the TCGA-THCA data set, and carried out standardized correction on the FPKM data through R software for differential analysis. Next, identify the specific conditions of DEGs through the "LIMMA" package by comparison between data of 58 adjacent nontumorous tissue samples and these of 510 THCA tissues. Based on *p < 0*.*05* and |log_2_ fold change (FC)| ≥1, we can find RBPs with different expressions. Then through R software, two kinds of enrichment analysis of RBPs with very obvious differences were carried out, namely the Kyoto Encyclopedia of Genome Genomics Database (KEGG) enrichment analysis and Gene Ontology (GO) enrichment analysis (*p*<0.05).

### 2.3 Protein-protein interaction (PPI) network construction and module analysis

To predict the PPI protein network of differentially expressed TCGA-THCA RBPs and to analyze the degree of interaction between proteins, we used the online website STRING (https://string-db.org/) [48–51]. Next, to make the PPI network visualized, we used Cytoscape software (v3.7.2) and then MCODE plug-in included in the Cytoscape software to expand the very critical sections of the PPI network survey. We defined advanced options as K-Core = 2, Node Score cutoff = 0.2, and degree cutoff = 2.

### 2.4 Prognosis-related RBPs selection

We used univariate Cox regression to examine the expression levels of DEGs in the TCGA dataset (*p*<0.05 as statistical significance), and then evaluated the prognostic value of RBPs and survival time, and finally determined the RBPs related to overall survival time. Also, the Kaplan Meyer plotter (https://kmplot.com/analysis/) was used to further verify the prognostic value of various RBPs [52]. The RBPs with *p < 0*.*05* were considered to be true prognostic RBPs.

### 2.5 Prognostic model construction and evaluation

From the prognostic-related RBPs genes obtained, we formulated a risk signature based on multivariate Cox proportional hazard regression analysis on the entire TCGA data (training set). The risk score of each sample in this model can be calculated by the following formula, which was:

$$Risk\;Score=\sum_{i=1}^{n}Expi\;\beta i$$

The β in this formula refers to the regression coefficient, and *Exp* indicates the gene expression value. Moreover, the model built by the “survival” package can estimate the performance of the prognostic model by the area under the (AUC) of the receiver operating characteristic (ROC) curve with optimal cut-off value. After the construction of the prognostic model, the entire TCGA data were randomly divided into two subsets for cross-validation. Finally, we also used the “rms” package to estimate the probability of overall survival (OS) occurrence and also drew a nomogram to predict 3- and 5-year OS for THCA patients. The calibration curve of the nomogram compared the predicted OS with the observed OS.

### 2.6 Analysis of key RBPs

To further identify the independent prognostic parameters in the model, we obtained the immunohistochemical results of thyroid cancer through the human protein atlas (HPA; https://www.proteinatlas.org/) [53–55]. cBioportal for Cancer Genomics was explored to investigate the genetic alterations of the prognostic genes in the gene signature. Besides, we use the data information in the TIMER database (https://cistrome.shinyapps.io/timer/) [56, 57], the correlation between RBP and tumor immune infiltration (including CD8+ T cells, neutrophils, dendritic cells, B cells, CD4+ T cells, and macrophages) was studied in depth.

### 2.7 Statistical analysis

In this study, we used R software (version 3.6.3) to explore the whole process. We used the R software packages "ggsignif", "ggpubr" and "ggplot2" to make box plots and quantitative statistical studies of differential expression. In R, we performed multivariate and univariate Cox analysis. The ROC analysis was calculated by the "survivalROC" R package. *p <* 0.05 and considered statistically significant.

## 3. Result

### 3.1 Identification of differentially expressed RBPs and functional enrichment analysis in THCA

**Fig 1:**
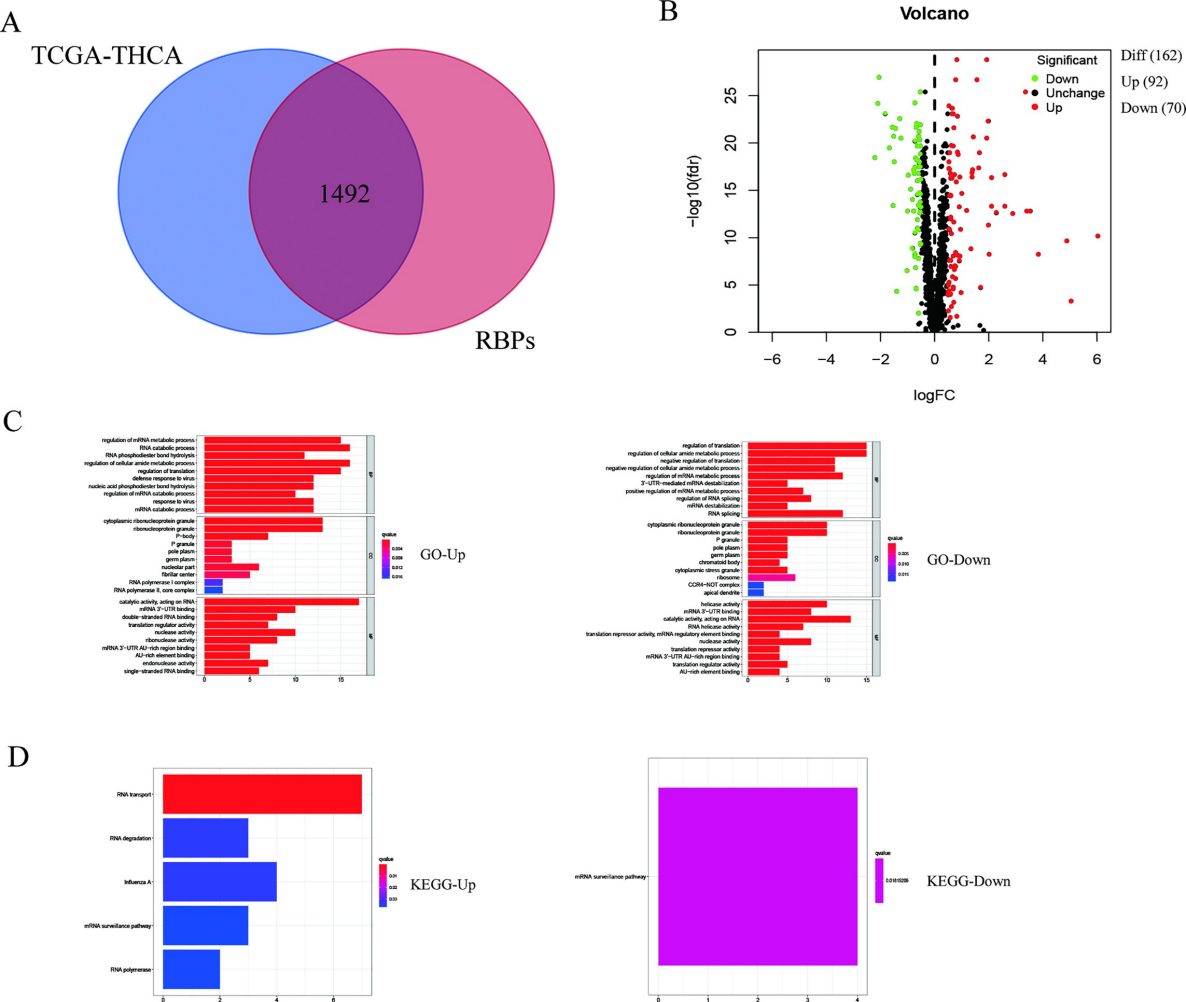
Identification of differentially expressed RBPs-related genes and functional enrichment analysis in THCA. (A) Venn diagrams of RBPs in THCA. Overlapping area means the 1492 genes are in both TCGA-THGA gene list (blue) and RBPs gene list (red); (B) Differentially expressed genes (DEGs) analysis of DEGs; (C) GO enrichment analysis of DEGs; (D) KEGG enrichment analysis of DEGs, p < 0.05.

The intersection of the collected 1542 RBPs data sets and THCA data sets shows that there were 1492 RBPs related genes in THCA (Fig 1A). Then we did a difference analysis of these 1492 THCA RBPs between adjacent normal tissues vs thyroid cancer samples and 162 genes were identified with significant differences (S1 Table). Among them were 70 down-regulated genes and 92 up-regulated genes as displayed in Fig 1B. To assess the molecular mechanism and potential role of RBPs, we divided them into two parts according to their expression ability, and then by using R software we accomplished GO and KEGG enrichment analysis on these two groups, respectively (Fig 1C and 1D). Mostly, RBPs work through multiple RNA-related functions in this result (the list is shown in S2 Table).

### 3.2 PPI network construction and key gene screening

**Fig 2:**
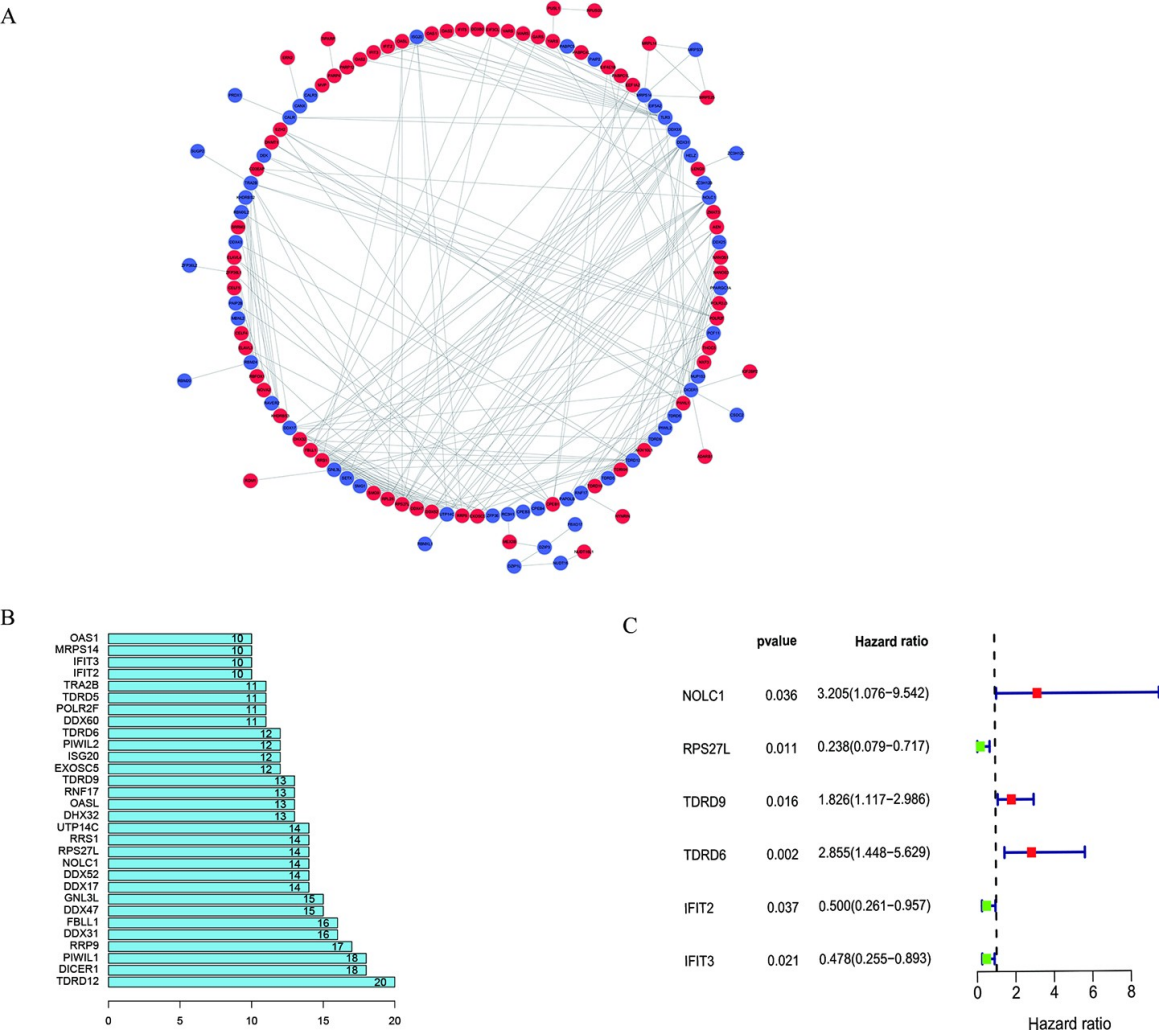
PPI network and key prognostic gene. (A) PPI network for RBPs. blue: down-regulated genes, red: up-regulated genes; (B) Top 30 nodes in PPI network with the number of interactions; (C) Prognostic-related genes in TCHA.

**Fig 3:**
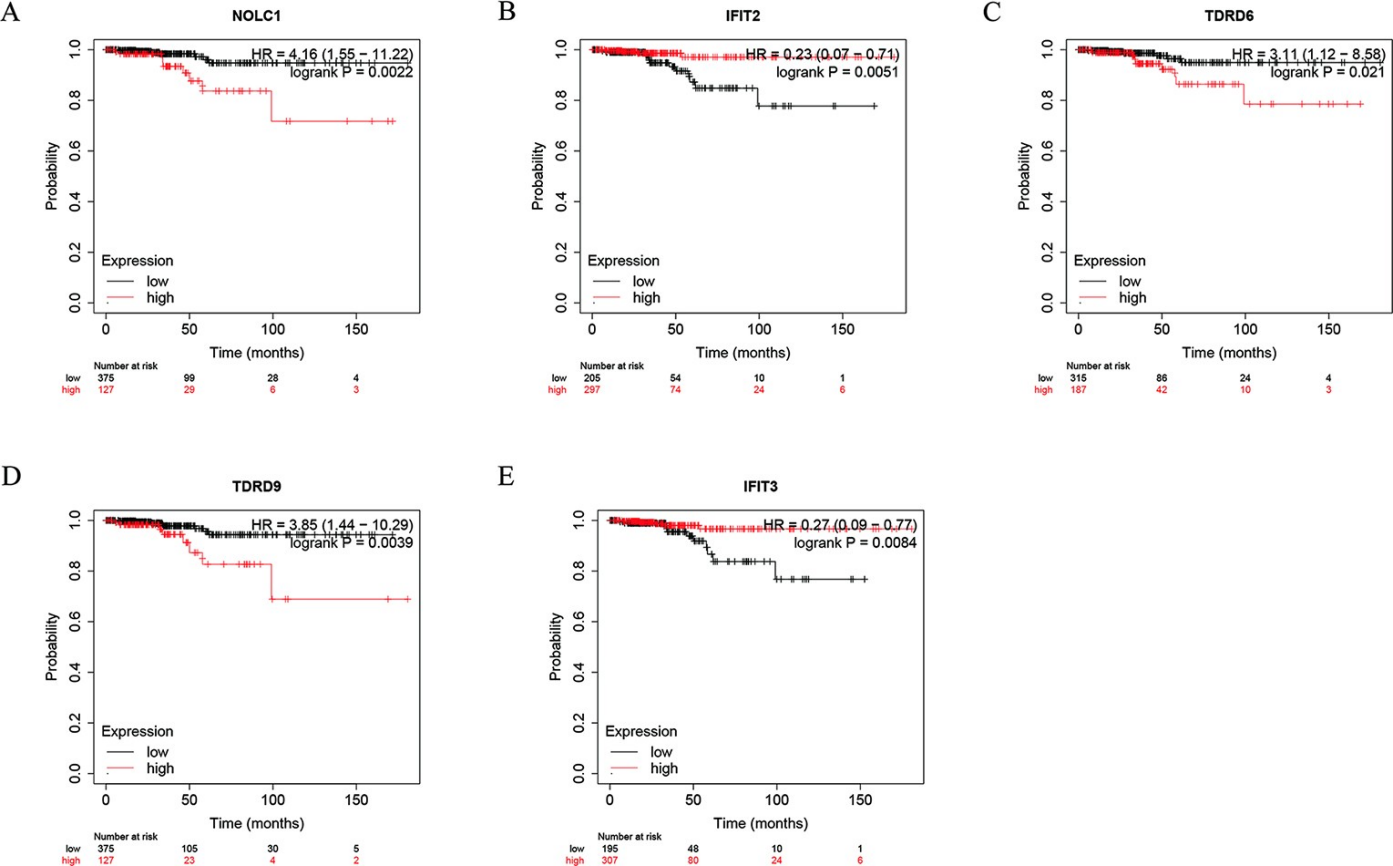
The Kaplan-Meier plotter analysis. (A) NOLC1; (B) IFIT2; (C) TDRD6; (D) TDRD9; (E) IFIT3.

To better understand these DEGs and seek for the potential key RBPs as biomarker candidates in the development of thyroid cancer, we constructed a PPI network among DEGs through the online website STRING, and visualized it through Cytoscape software. As depicted in Fig 2A, this PPI network has a total of 408 edges and 133 nodes. Also, we calculated the number of interactions between each node and visualized the first 30 nodes as candidate key RBPs for further examination, as presented in Fig 2B. To identify the genes related to the prognosis of thyroid cancer, we utilized univariate Cox regression analysis and found that *NOLC1* (*p =* 0.036), *RPS27L* (*p =* 0.011), *TDRD9* (*p =* 0.016), *TDRD6* (*p =* 0.002), *IFIT2* (*p =* 0.037), and *IFIT3* (*p =* 0.02) were prominently involved in the prognosis of thyroid cancer (Fig 2C; Table 1). Additionally, we determined the prognostic ability of these 6 RBP-coding genes through the Kaplan-Meier plotter (Fig 3). Among them, there was no data for *RPS27L* at the Kaplan-Meier plotter database. These outcomes revealed that these 6 genes were significantly related to prognosis.

**Table 1:** Univariate Cox regression analysis results.

ID	HR	HR.95L	HR.95H	p value
NOLC1	3.20	1.07	9.54	0.036
RPS27L	0.23	0.078	0.71	0.010
TDRD9	1.82	1.11	2.98	0.016
TDRD6	2.85	1.44	5.62	0.0024
IFIT2	0.49	0.26	0.95	0.036
IFIT3	0.47	0.25	0.89	0.020

### 3.3 Prognosis-related risk score model construction and validation

**Fig 4:**
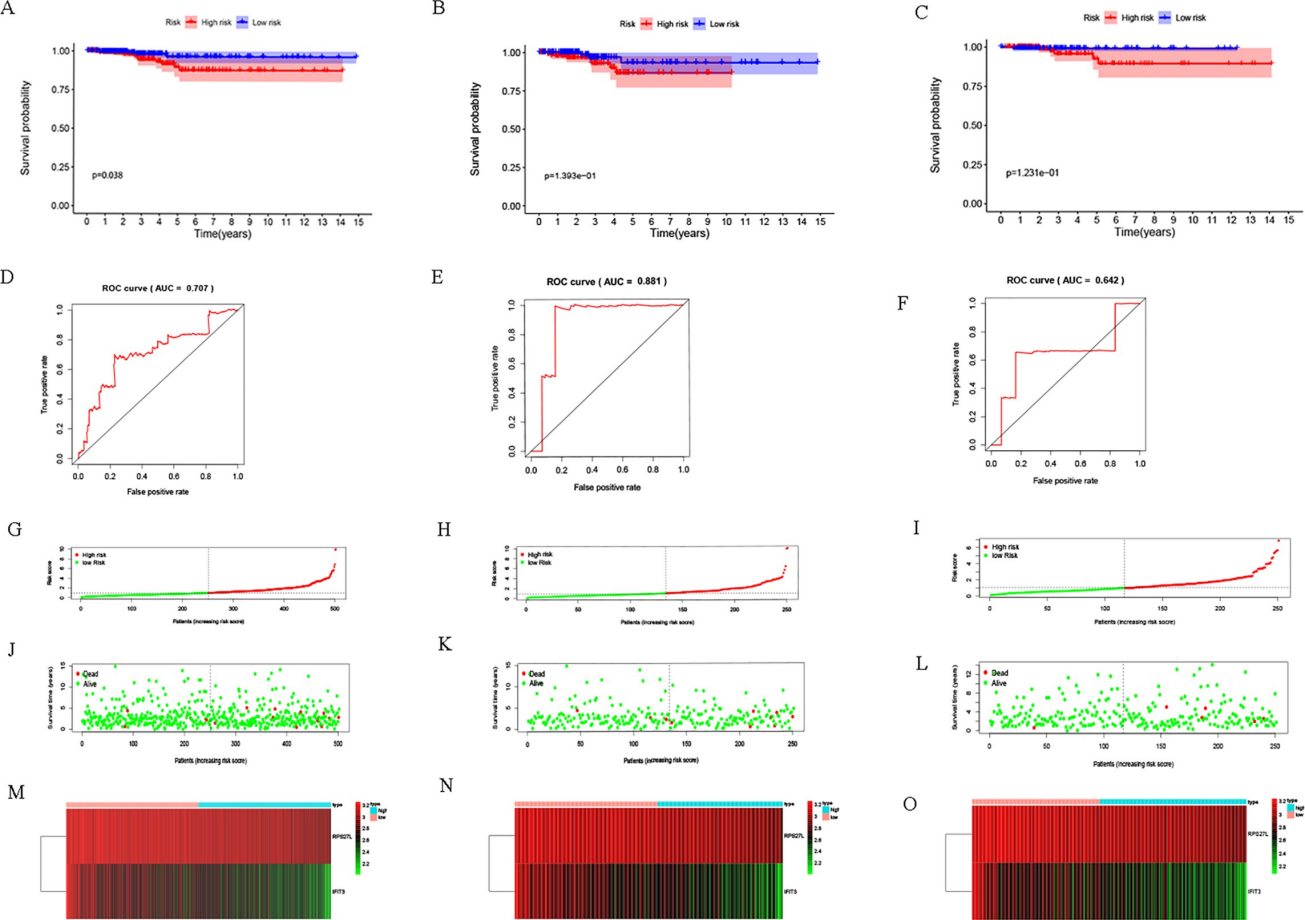
Risk score analysis of the prognostic model. (A) The Kaplan–Meier survival curves in the training set; (B-C) The Kaplan–Meier survival curves in two validation sets; (D) ROC analysis in the training set; (E-F) ROC analysis in the two validation sets; (G) Distribution of risk scores in the training set; (H-I) Distribution of risk scores in the validation sets; (J) Distribution of survival status of patients in different groups in the training set; (K-L) Distribution of survival status of patients in different groups in the two validation sets; (M) Heat map of 2 genes’ expression along with risk scores in the training set; (N-O) Heat map of 2 genes’ expression along with risk scores in the two validations sets.

Through Multivariate Cox analysis on the entire TCGA training set, we identified 2 RBP-coding genes (*RPS27L* and *IFIT3*) to construct a predictive model. To predict the model and to carry out survival analysis, we divided THCA patients into two groups by using an optimal cut-off value of risk scores in the training set, namely the high-risk group and low-risk group. The Kaplan–Meier survival curve demonstrated that the high-risk subgroup was significantly related to poor survival (*p* = 0.038; Fig 4A). By completing the time-related ROC investigation, we can more accurately judge the prognostic value of the 2-gene model (AUC = 0.707; Fig 4D). Further, we studied the risk score distribution (Fig 4G), distribution of patients with survival status (Fig 4J) and expression heat map (Fig 4M) with the 2-RBP gene biomarker within the high-risk and low-risk subgroups in the training set.

To test the predictive power of the model, we randomly allocated two subsets from the entire TCGA data and evaluated the risk scores and optimal critical values using the same formula. The high-risk group and the low-risk group in each validation subset were divided accordingly and high-risk group presented poor prognosis (Fig 4B and 4C). The AUCs in the validation subsets were 0.881 and 0.642, respectively (Fig 4E and 4F). Risk score distribution and survival status distribution are exposed in Fig 4H, 4I, 4K and 4L. The heatmaps disclose the expression of the 2 genes relatively increased along with the rise of risk scores (Fig 4N and 4O). Together, these findings implied that the 2-gene model had a good performance on the prediction of OS in THCA patients.

**Fig 5:**
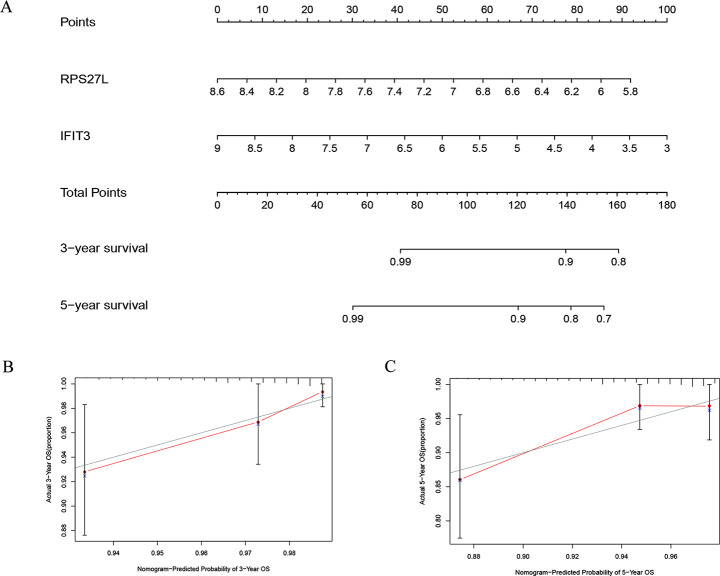
Nomogram and calibration plots of RBPs. (A) Nomogram to predict 3- and 5-year OS in the TCGA cohort; (B, C) Calibration plots of the nomogram to predict OS at 3 and 5 years.

To construct a quantitative model for THCA prognosis, we combined the two-RBP marker to build a nomogram plot (Fig 5A) for visualization of the performance of the 2-gene prognostic model by the multivariate Cox regression in the entire TCGA data. Calibration plots also revealed that the model had a good performance in estimation of 3-year or 5-year survival of THCA patients (Fig 5B and 5C) with a harmonious consistency (C-index = 0.67) between the predicted and observed survival.

### 3.4 Clinical correlation analysis

**Fig 6:**
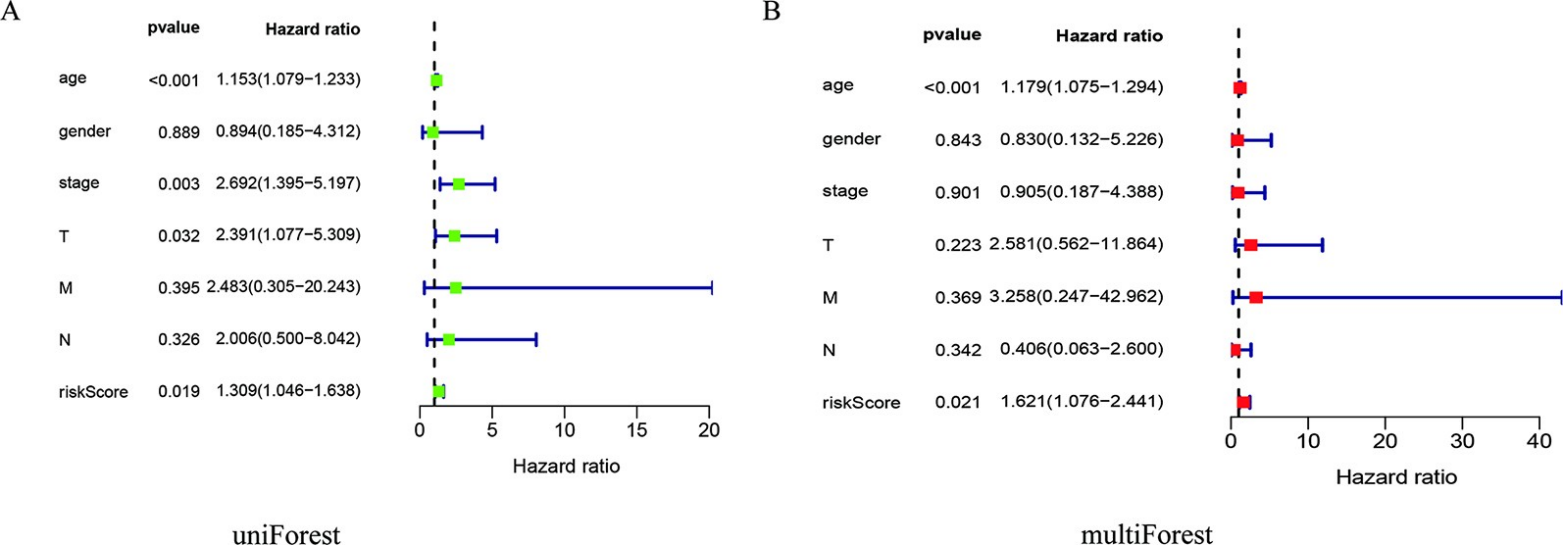
Univariate and multivariate Cox analysis. (A) Univariate Cox analysis; (B) multivariate Cox analysis.

**Fig 7:**
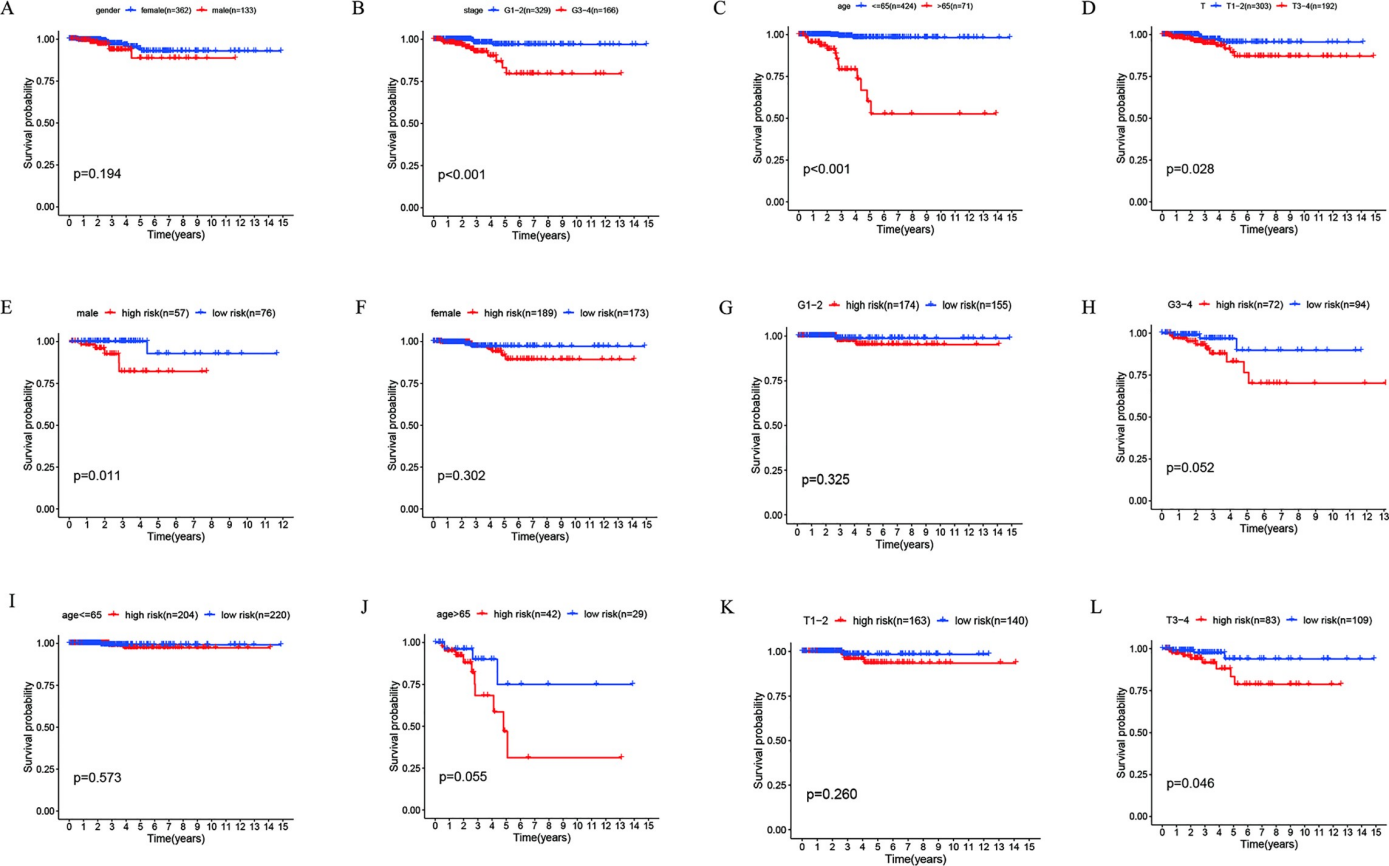
Clinical correlation analysis.

To assess the correlation between THCA patients and clinics, we found that age (*p <* 0.001), stage (*p =* 0.003), T (*p =* 0.032), and risk score (*p =* 0.019) were all related to prognosis through a univariate Cox analysis (Fig 6A). Multivariate Cox analysis exhibited that age (*p <* 0.001) and riskScore (*p =* 0.021) were related to prognosis (Table 2; Fig 6B). Besides, we used the R software packages "survival" and "survminer" to evaluate, and the results indicated that stage (G3-4), age (> 65), and T (T3-4) were all related to poor prognosis (Fig 7B–7D). Further, we verified and divided THCA into high-risk and low-risk subgroups. The outcomes of the study disclosed that the high-risk was from the male group (*p =* 0.011), and the T3-4 group (*p =* 0.046) was associated with a worse prognosis (Fig 7E and 7L).

**Table 2:** The prognostic effect of different clinical parameters.

	Univariate analysis				Multivariate analysis			
	HR	HR.95L	HR.95H	p value	HR	HR.95L	HR.95H	p value
age	1.15	1.08	1.23	2.81E-05	1.17	1.07	1.29	0.00046
gender	0.89	0.18	4.31	0.88	0.83	0.13	5.22	0.84
stage	2.69	1.39	5.19	0.003	0.90	0.18	4.38	0.90
T	2.39	1.08	5.30	0.032	2.58	0.56	11.86	0.22
M	2.48	0.30	20.24	0.39	3.25	0.24	42.96	0.36
N	2.01	0.50	8.04	0.32	0.40	0.063	2.60	0.34
riskScore	1.31	1.05	1.63	0.019	1.62	1.07	2.44	0.021

### 3.5 Validate and analyze the RBP-coding genes in the model

**Fig 8:**
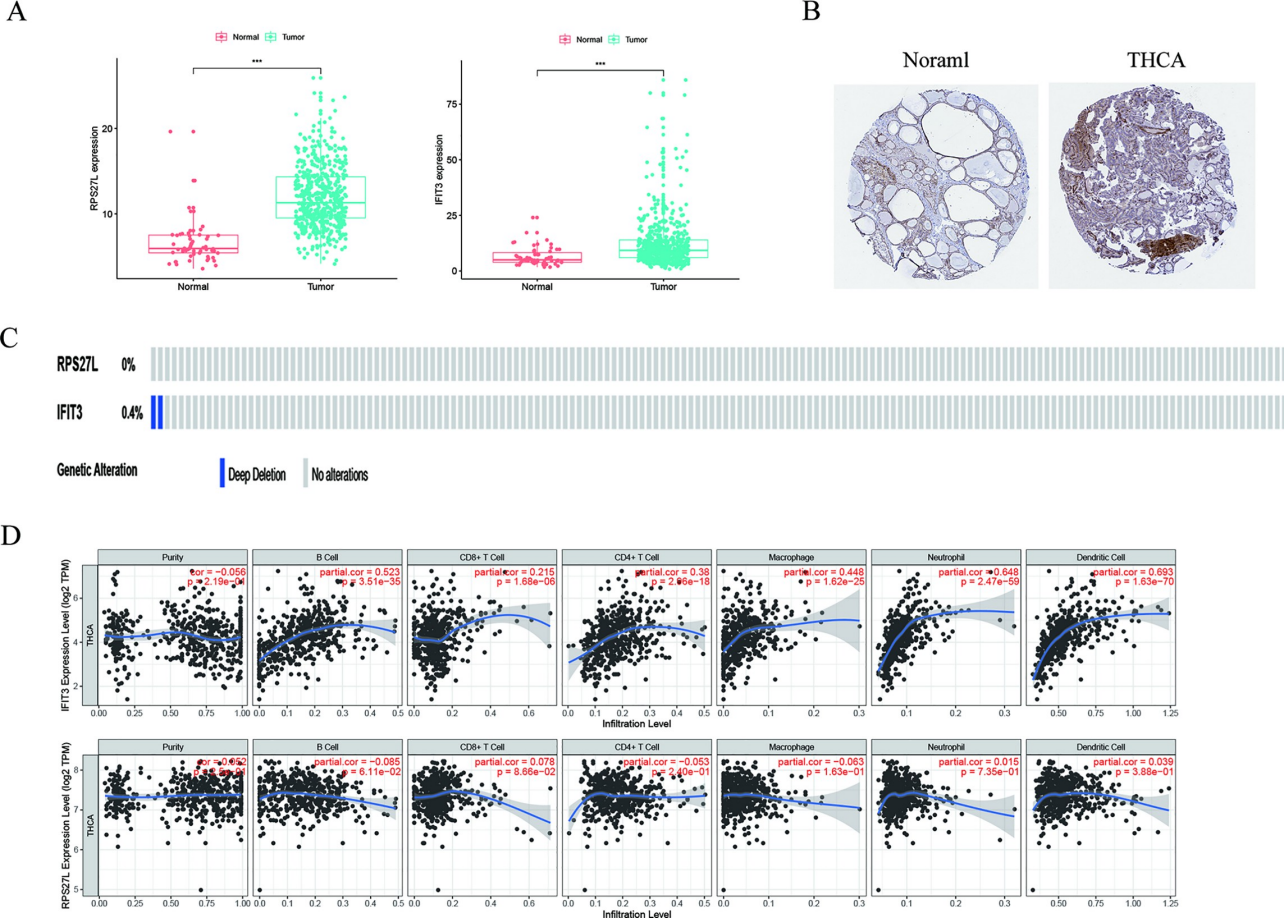
Validate and analyze the RBP-coding genes. (A) RPS27L and IFIT3 were highly expressed in THCA (TCGA, p***<0.001); (B) IFIT3 was highly expressed in THCA (HPA); (C) RPS27L and IFIT3 mutations were very low; (D) Correlation analysis between gene expression and immune infiltrates.

**Fig 9:**
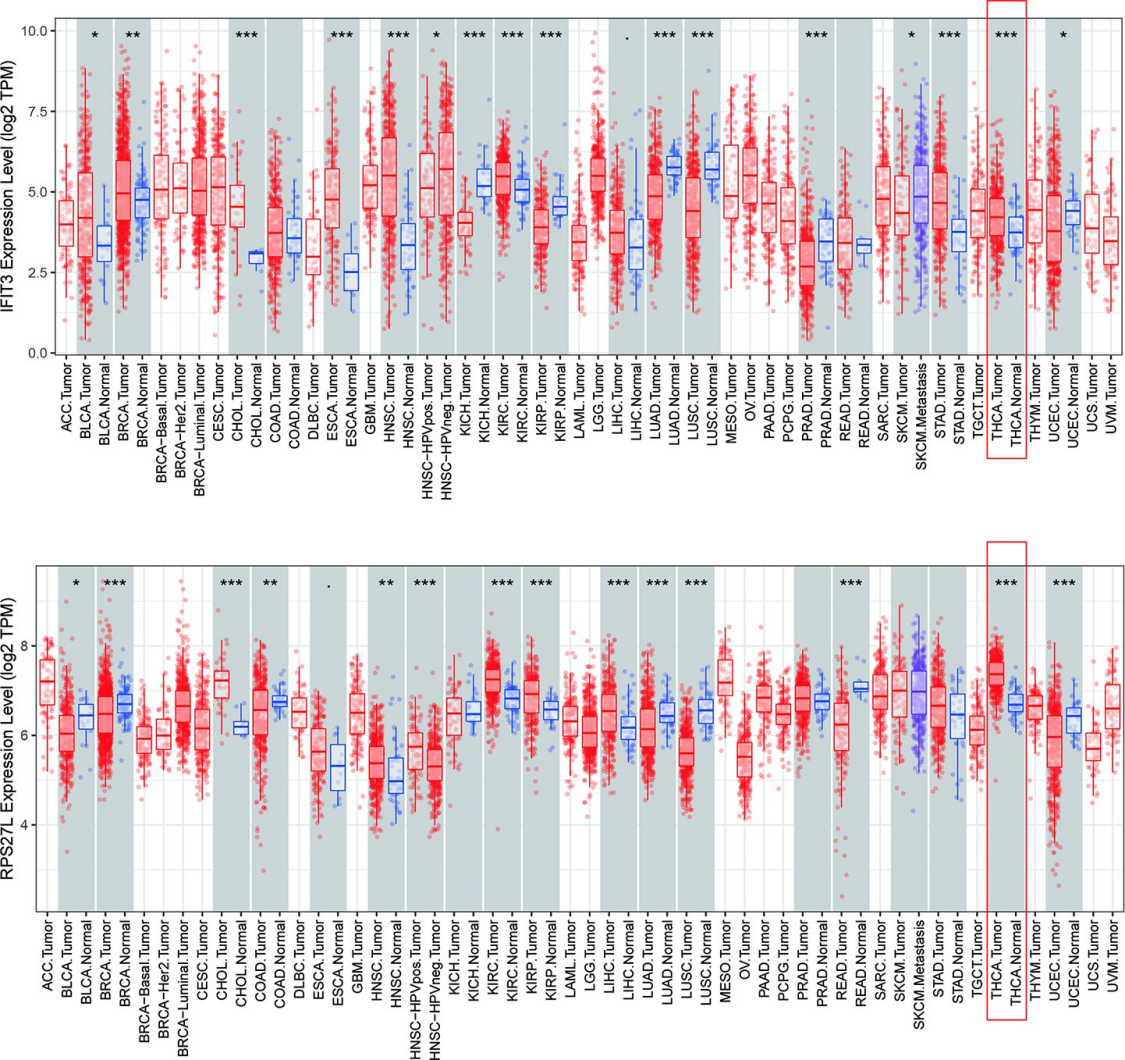
The expression of the two predictive genes in cancers. Data were from the TIMER database. p*<0.05; p**<0.01; p***<0.001.

We examined the expression of RBP-encoding genes in THCA through R software, and the results displayed that *RPS27L* and *IFIT3* were highly expressed in TCGA-THCA patients (Fig 8A, p***< 0.001). It was verified using the HPA database, and the findings exhibited that positive *IFIT3* in THCA was much stronger compared with normal tissues (Fig 8B). However, there was no immunohistochemistry result for RPS27L in the HPA database. Later mutation analysis was carried out on *RPS27L* and *IFIT3* through the cBioPortal online tool, and the results proved that the mutation frequency of both was very low (Fig 8C). Also, through the TIME online tool, we found that *IFIT3* has a positive correlation with Macrophage (r = 0.448, p***< 0.001), Neutrophil (r = 0.648, p***< 0.001), CD4 + T cell (r = 0.38, p** < 0.01), B cell (r = 0.523, p** < 0.01), Dendritic cell (r = 0.693, p*** < 0.001) and CD8 + T cell (r = 0.215, p** < 0.01) immune infiltration levels of THCA (Fig 8D). Through TIME online tool, the results indicated that both *RPS27L* and *IFIT3* were highly expressed in tumors (Fig 9).

## 4. Discussion

Thyroid tumors are the most common endocrine tumors. Many researchers reported that abnormal expression of RBPs can cause several illness including cancer. In previous studies, it can be found that the RNA binding proteins PTRF and FNDC3B have been identified as potential prognostic biomarkers for glioblastoma [58]. Three RBP genes (*NOVA1*, *EZH2*, and *RBM24*) were identified as central genes related to the prognosis of head and neck squamous cell carcinoma (HNSCC) [59]. RBP has also been found to play a very important role in lung cancer [60]. Therefore, some therapeutic drugs have been developed to target RBPs to treat some specific cancer [45–47]. Thus, we conducted this study to further explore the role of RBP in thyroid cancer.

We collected 58 adjacent non-tumor tissue samples and 510 THCA tissues from the TGCA database. Extraction of RBP-related genes and analysis of differential expression was carried out, and 162 significantly different RBP-related genes were identified. the data demonstrated that there were 70 down-regulated genes and 92 up-regulated genes. Further we explored the KEGG and GO enrichment investigations for up-regulated genes and noticed that RNA catabolism process, the cellular amide metabolism process, virus defense reaction, cell regulation, amide metabolism process regulation, nucleic acid phosphodiester bond hydrolysis, translation regulation, RNA phosphodiester bond hydrolysis, catabolism regulation process, response to virus, mRNA catabolic process, P-body, ribonucleoprotein granule, cytoplasmic ribonucleoprotein granule, cytoplasmic stress granule, P granule, pole plasm, germplasm, nucleolar part, RNA polymerase II, core complex, fibrillar center, catalytic activity, acting on RNA, nuclease activity, translation regulator activity, double-stranded RNA binding, mRNA 3’-UTR binding, ribonuclease activity, mRNA 3’-UTR AU-rich region binding, AU-rich element-binding, exonuclease activity, single-stranded RNA binding, RNA transport, RNA degradation, Influenza A, mRNA surveillance pathway, and RNA polymerase were enriched. On the other hand, we performed same enrichment study for down-regulated genes and findings showed that mRNA metabolism process Regulation, cellular amide metabolism process regulation, translation regulation, positive regulation of mRNA metabolism, 3’-UTR-mediated mRNA instability and instability, translation negative regulation, regulation of RNA splicing, mRNA instability, cellular amide metabolism process negative regulation, RNA splicing, cytoplasmic ribonucleoprotein granule, ribonucleoprotein granule, P granule, pole plasm, germplasm, chromatoid body, cytoplasmic stress granule ribosome, CCR4-NOT complex, apical dendrite, helicase activity, mRNA 3’-UTR binding, catalytic activity, acting on RNA, RNA helicase activity, translation repressor activity, mRNA regulatory element-binding, AU-rich element-binding, mRNA 3’-UTR AU-rich region binding, translation repressor activity, translation regulator activity, nuclease activity, and mRNA surveillance pathway were enriched. Our results proved that the expectation of enriched pathways of a group of RBPs genes are RNA-related functions. Some RNA-related functions listed on the top might be critical in thyroid cancer development but need further study.

Later, we did PPI network integration to assess those RBPs who are likely to have major impact on the RBPs network in thyroid cancer. The changes in the expression level of those RBPs with more interactions might influence a larger amount of downstream pathways. Next, we conducted univariate Cox regression analysis on these genes and identified 6 genes (*NOLC1*, *RPS27L*, *TDRD9*, *TDRD6*, *IFIT2*, and *IFIT3*) involved in prognosis. We further verified these prognostic genes through the online website Kaplan-Meier plotter. Based on their correlation with prognosis, they might work as potential biomarkers or play an important part in the development of thyroid cancer. We have launched a new round of selection for the key RBP related to prognosis. Meanwhile, we build a risk model based on the characteristics of dual RBP genes (*RPS27L* and *IFIT3*) that can predict the prognosis of THCA. Moreover, to begin a new round of prognostic performance of the judging model, we carried out an ROC study on the time dependence of this model with a decent effectivity (AUC = 0.707). To assess the predictive ability of this model, we divided THCA patients into two groups including a high-risk group and a low-risk group and completed survival and clinical correlation examination, respectively. We observed a strong correlation between high-risk subgroups and low survival rates (*p* = 0.038), which suggested that those THCA patients with high risk predicted by the 2-gene model are prone to a worse prognosis. The better performance of this model in the validation subsets supported its comparable prognostic ability. Also, the high-risk subgroups in both the male group (*p* = 0.011) and the T3-4 group (*p* = 0.046) were associated with a worse prognosis. Finally, we explored the genes that make up the model. TCGA data analysis showed that both genes were highly expressed in tumors. HPA database revealed that IFIT3 was highly expressed in THCA. TIMER database results displayed that both *RPS27L* and *IFIT3* were highly expressed in tumors. The mutation investigation outcomes concluded that both genes were very low mutation frequency, indicating their dysregulation might be mediated by other means. However, the stable mutation status of *RPS27L* and *IFIT3* together with their high expression in tumor tissues makes them potential biomarkers as well as therapeutic targets in thyroid cancer. Furthermore, we found that *IFIT3* has a positive correlation with Macrophage (r = 0.448, *p* < 0.001), Neutrophil (r = 0.648, *p* < 0.001), CD4 + T cell (r = 0.38, *p* < 0.01), B cell (r = 0.523, *p* < 0.01), Dendritic cell (r = 0.693, *p* < 0.001) and CD8 + T cell (r = 0.215, *p* < 0.01) immune infiltration levels of THCA. Although we have not found any report about how *IFIT3* works in cancer, based on the significant association of *IFIT3* and prognosis of thyroid cancer patients, we think its insight should be further studied. These correlations with multiple immune subsets suggest the function of *IFIT3* might be associated with the cancer microenvironment and immune system.

However, we faced some limitations, such as the interesting finding is the individual RBP gene was associated with a better prognosis, while the established model was associated with a worse prognosis. This may be the direction we need to study next such as the lack of verification of the 2-gene predictive model by another independent cohort and the wet experiments of this model for reliability.

Overall, we systematically examined the role of RBPs prognosis of THCA and provided a new perspective on the role of RBPs in the THCA. Mainly, this 2-gene model containing *RPS27L* and *IFIT3* may provide us with great prognostic indicators for the development of THCA. In addition, our study of *IFIT3* implies its great potential in thyroid cancer progressing which has not been reported yet, and these RBPs especially *RPS27L* and *IFIT3* may also be used in clinical adjuvant therapy.

## Supporting information

S1 Table Identification of differentially expressed RBPs. (DOCX)

S2 Table GO and KEGG pathway analysis results. (DOCX)
