# Supplementary material for: Integrated analysis of RNA-binding proteins in thyroid cancer
Source: PLoS One. 2021 Mar 12;16(3):e0247836. doi: 10.1371/journal.pone.0247836 (PMC7954316; doi:10.1371/journal.pone.0247836)
Supplement: S1 Table — (DOCX) [file pone.0247836.s001.docx]

**S1 Table. Identification of Differentially Expressed RBPs.**

| **gene** | **conMean** | **treatMean** | **logFC** | **pValue** | **fdr** |
| --- | --- | --- | --- | --- | --- |
| DEK | 47.76347 | 33.52348 | -0.51074 | 5.38E-18 | 7.18E-17 |
| ZNF579 | 3.608335 | 5.893583 | 0.707812 | 3.84E-13 | 2.21E-12 |
| RBFOX1 | 0.012483 | 0.369647 | 4.88809 | 4.89E-11 | 2.18E-10 |
| L1TD1 | 0.15187 | 0.052643 | -1.52853 | 4.89E-15 | 4.01E-14 |
| ELAVL3 | 0.005962 | 0.085235 | 3.837651 | 1.64E-09 | 5.96E-09 |
| ELAVL4 | 0.013726 | 0.026962 | 0.974015 | 3.07E-05 | 6.07E-05 |
| PUSL1 | 2.003717 | 3.33426 | 0.734688 | 1.59E-18 | 2.28E-17 |
| SMG9 | 2.92805 | 4.4028 | 0.588481 | 3.67E-18 | 4.99E-17 |
| TDRD9 | 5.232691 | 1.859099 | -1.49295 | 4.76E-20 | 9.54E-19 |
| ZC3H12B | 0.596365 | 0.394975 | -0.59443 | 3.00E-12 | 1.57E-11 |
| ZC3H12C | 3.823549 | 2.223775 | -0.7819 | 4.22E-19 | 6.67E-18 |
| GARS | 14.94673 | 24.51021 | 0.713553 | 4.13E-24 | 2.34E-22 |
| CD3EAP | 0.806165 | 1.184853 | 0.555561 | 3.82E-19 | 6.18E-18 |
| RDM1 | 0.056081 | 0.176325 | 1.652642 | 4.38E-21 | 1.15E-19 |
| DZIP1L | 0.810408 | 0.487086 | -0.73447 | 1.23E-09 | 4.60E-09 |
| DICER1 | 7.341492 | 4.657991 | -0.65637 | 2.92E-20 | 6.11E-19 |
| SAMD4A | 1.829817 | 4.835237 | 1.401887 | 4.21E-19 | 6.67E-18 |
| RNASEK | 2.870882 | 4.133474 | 0.52586 | 4.71E-20 | 9.54E-19 |
| APOBEC2 | 0.151343 | 0.384788 | 1.346244 | 3.75E-10 | 1.53E-09 |
| APOBEC3H | 0.402496 | 1.242083 | 1.625715 | 2.56E-19 | 4.25E-18 |
| EIF5A2 | 0.94511 | 0.604218 | -0.64541 | 6.53E-22 | 2.12E-20 |
| FBXO17 | 0.538968 | 0.272052 | -0.98632 | 1.74E-18 | 2.47E-17 |
| SPATS2L | 8.011926 | 13.97926 | 0.803067 | 1.05E-17 | 1.33E-16 |
| SPATS2 | 3.977951 | 6.982676 | 0.811754 | 1.18E-32 | 1.51E-29 |
| PARP12 | 3.946212 | 5.956834 | 0.594077 | 3.50E-16 | 3.53E-15 |
| TIPARP | 12.3706 | 21.04613 | 0.766639 | 3.45E-08 | 1.04E-07 |
| NUP153 | 8.891505 | 6.104913 | -0.54246 | 2.25E-16 | 2.41E-15 |
| TDRD5 | 0.401343 | 0.225734 | -0.83021 | 3.07E-10 | 1.27E-09 |
| POLR2J3 | 0.463259 | 0.692219 | 0.579407 | 0.019791 | 0.026589 |
| SUGP2 | 13.3124 | 8.962584 | -0.57078 | 1.96E-19 | 3.41E-18 |
| AZGP1 | 0.227579 | 0.086442 | -1.39656 | 2.47E-05 | 4.95E-05 |
| NYNRIN | 6.296278 | 8.915852 | 0.501873 | 6.68E-05 | 0.000125 |
| RRP9 | 10.32521 | 14.86036 | 0.525299 | 3.18E-19 | 5.21E-18 |
| PRDX1 | 372.4998 | 253.5594 | -0.55492 | 1.74E-16 | 1.91E-15 |
| TDRKH | 3.244627 | 5.050644 | 0.638414 | 4.05E-18 | 5.46E-17 |
| OASL | 1.051254 | 1.503349 | 0.516069 | 0.003347 | 0.005095 |
| OAS1 | 2.454154 | 3.92285 | 0.676677 | 7.77E-06 | 1.68E-05 |
| OAS2 | 2.831274 | 4.3075 | 0.6054 | 3.63E-08 | 1.09E-07 |
| OAS3 | 2.451883 | 3.923472 | 0.678241 | 1.36E-05 | 2.80E-05 |
| MYEF2 | 0.553371 | 1.639253 | 1.566719 | 7.20E-30 | 2.07E-27 |
| RNASE10 | 0.015201 | 0.091875 | 2.595505 | 1.41E-18 | 2.07E-17 |
| CALR | 858.3946 | 560.4662 | -0.61501 | 2.87E-12 | 1.50E-11 |
| CANX | 246.2381 | 158.2988 | -0.6374 | 2.18E-13 | 1.29E-12 |
| CALR3 | 0.025179 | 0.016744 | -0.58856 | 0.006815 | 0.009868 |
| MBNL2 | 18.03428 | 11.66414 | -0.62866 | 2.79E-20 | 5.94E-19 |
| RPS27L | 6.633198 | 11.95553 | 0.849901 | 1.91E-25 | 1.53E-23 |
| SMAD1 | 2.954397 | 2.086092 | -0.50206 | 7.24E-15 | 5.57E-14 |
| SMAD9 | 20.78614 | 7.005475 | -1.56907 | 3.63E-24 | 2.16E-22 |
| ADAD2 | 0.013712 | 0.024209 | 0.82016 | 0.015663 | 0.02136 |
| ADARB1 | 1.360894 | 2.038281 | 0.582799 | 1.32E-13 | 8.11E-13 |
| IGF2BP2 | 3.131987 | 12.34878 | 1.979219 | 7.25E-25 | 5.20E-23 |
| TDRD10 | 1.2628 | 2.540236 | 1.008336 | 2.03E-16 | 2.20E-15 |
| MOV10L1 | 0.041868 | 0.068427 | 0.708727 | 0.000379 | 0.000649 |
| AFF2 | 0.091149 | 0.392865 | 2.107734 | 3.26E-18 | 4.48E-17 |
| WARS | 39.2518 | 67.20849 | 0.775885 | 9.04E-07 | 2.17E-06 |
| RBM46 | 0.094113 | 0.053441 | -0.81645 | 9.49E-16 | 8.85E-15 |
| RBM20 | 0.434276 | 0.263615 | -0.72018 | 2.81E-09 | 9.71E-09 |
| GNL3L | 4.588155 | 2.69216 | -0.76915 | 2.14E-14 | 1.48E-13 |
| DDX52 | 1.774737 | 3.059989 | 0.785922 | 7.61E-30 | 2.07E-27 |
| LENG9 | 0.894872 | 2.03622 | 1.186141 | 2.03E-14 | 1.41E-13 |
| TDRD6 | 1.280849 | 0.47147 | -1.44186 | 5.28E-24 | 2.87E-22 |
| ATXN1L | 7.988998 | 5.590257 | -0.5151 | 2.49E-16 | 2.62E-15 |
| NXF3 | 0.009548 | 0.057423 | 2.588287 | 6.64E-15 | 5.14E-14 |
| RRS1 | 6.277829 | 9.586058 | 0.610672 | 5.14E-22 | 1.84E-20 |
| PIWIL1 | 0.244972 | 2.839054 | 3.534719 | 2.29E-14 | 1.55E-13 |
| PIWIL2 | 0.335889 | 0.182268 | -0.88192 | 6.35E-17 | 7.51E-16 |
| ACO1 | 9.400428 | 15.08203 | 0.682032 | 2.45E-09 | 8.62E-09 |
| PCBP4 | 4.991857 | 7.506333 | 0.588532 | 1.74E-13 | 1.04E-12 |
| PAIP2 | 23.7316 | 16.05509 | -0.56378 | 1.27E-23 | 6.39E-22 |
| PAIP2B | 2.988338 | 2.060756 | -0.53617 | 8.08E-15 | 6.01E-14 |
| RNASE1 | 28.10132 | 39.76132 | 0.500728 | 3.52E-05 | 6.90E-05 |
| RNASE2 | 0.156081 | 0.67219 | 2.106574 | 6.07E-15 | 4.80E-14 |
| RNASE3 | 0.011176 | 0.045373 | 2.021416 | 1.49E-09 | 5.46E-09 |
| RBMXL1 | 5.35187 | 3.312997 | -0.69191 | 3.65E-24 | 2.16E-22 |
| RBMXL2 | 0.023788 | 0.01491 | -0.67394 | 1.24E-05 | 2.56E-05 |
| PPP1R10 | 28.47044 | 19.33815 | -0.55801 | 3.85E-14 | 2.52E-13 |
| TRA2B | 14.09347 | 9.74136 | -0.53283 | 1.89E-24 | 1.23E-22 |
| EIF3CL | 0.061198 | 0.111486 | 0.865296 | 2.30E-09 | 8.11E-09 |
| SRRM3 | 0.344282 | 0.904692 | 1.393835 | 2.76E-18 | 3.87E-17 |
| PIH1D3 | 0.079179 | 0.02471 | -1.68002 | 1.14E-21 | 3.44E-20 |
| CSDC2 | 0.304556 | 0.187081 | -0.70304 | 4.81E-08 | 1.42E-07 |
| DDX25 | 0.749099 | 0.180454 | -2.05353 | 2.51E-30 | 1.14E-27 |
| SMG1 | 5.267915 | 3.653021 | -0.52814 | 2.65E-14 | 1.79E-13 |
| EXOSC5 | 8.28909 | 12.13268 | 0.549613 | 3.36E-16 | 3.41E-15 |
| RC3H1 | 4.11861 | 2.746539 | -0.58454 | 4.23E-23 | 1.99E-21 |
| ZFP36 | 327.4369 | 160.5292 | -1.02838 | 1.09E-07 | 3.03E-07 |
| ZFP36L1 | 87.81544 | 171.0619 | 0.961972 | 2.49E-12 | 1.31E-11 |
| ZFP36L2 | 204.012 | 83.56018 | -1.28777 | 3.69E-25 | 2.79E-23 |
| NUDT16 | 13.85814 | 8.369393 | -0.72754 | 1.48E-22 | 5.92E-21 |
| NUDT16L1 | 16.90387 | 24.73713 | 0.549324 | 2.71E-12 | 1.42E-11 |
| THOC5 | 1.854162 | 3.327742 | 0.843776 | 3.30E-21 | 9.21E-20 |
| EZH2 | 0.814682 | 1.171939 | 0.524588 | 2.27E-12 | 1.21E-11 |
| ZMAT3 | 2.784217 | 10.60221 | 1.929022 | 2.22E-32 | 1.51E-29 |
| MVP | 16.32564 | 43.98973 | 1.430027 | 5.11E-23 | 2.32E-21 |
| EIF2AK3 | 8.555901 | 6.009694 | -0.50963 | 1.14E-10 | 4.89E-10 |
| KHDRBS2 | 6.772445 | 1.587588 | -2.09284 | 5.02E-27 | 6.84E-25 |
| KHDRBS3 | 0.561476 | 0.931547 | 0.730406 | 7.63E-08 | 2.19E-07 |
| RAVER2 | 2.990786 | 1.972242 | -0.60069 | 1.14E-18 | 1.71E-17 |
| PPARGC1A | 8.085034 | 2.290776 | -1.81942 | 7.26E-26 | 7.60E-24 |
| RBM24 | 0.614939 | 0.216077 | -1.5089 | 3.86E-23 | 1.88E-21 |
| VARS | 7.647959 | 11.86074 | 0.633047 | 9.71E-26 | 8.71E-24 |
| MIF4GD | 7.19266 | 11.53057 | 0.680866 | 6.86E-22 | 2.17E-20 |
| RNF17 | 0.051275 | 0.035506 | -0.53019 | 9.63E-11 | 4.19E-10 |
| RNASE11 | 0.000179 | 0.011683 | 6.031998 | 1.42E-11 | 6.83E-11 |
| EEF1A2 | 0.314147 | 3.325235 | 3.403946 | 2.28E-14 | 1.55E-13 |
| UTP14C | 8.174369 | 5.690267 | -0.52261 | 2.07E-15 | 1.78E-14 |
| DDX47 | 0.385919 | 0.561731 | 0.541581 | 7.84E-09 | 2.56E-08 |
| DDX3X | 50.70116 | 33.71498 | -0.58863 | 4.79E-09 | 1.61E-08 |
| DDX31 | 7.303993 | 4.988966 | -0.54994 | 1.23E-22 | 5.07E-21 |
| DNMT1 | 3.876909 | 5.85831 | 0.595578 | 4.10E-16 | 4.01E-15 |
| PCF11 | 8.406933 | 5.292877 | -0.66753 | 1.36E-24 | 9.26E-23 |
| MRPS14 | 14.54431 | 10.09737 | -0.52647 | 2.10E-28 | 4.06E-26 |
| ERN2 | 0.003868 | 0.02861 | 2.88702 | 4.49E-14 | 2.91E-13 |
| PIH1D2 | 2.10242 | 1.292045 | -0.70239 | 1.84E-20 | 4.18E-19 |
| POLR2F | 0.028224 | 0.047475 | 0.750269 | 3.04E-08 | 9.24E-08 |
| TLR3 | 2.273298 | 1.463301 | -0.63556 | 2.62E-16 | 2.71E-15 |
| DHX32 | 6.986424 | 10.05487 | 0.525268 | 9.29E-27 | 1.15E-24 |
| TDRD12 | 0.214558 | 0.151098 | -0.50588 | 1.09E-10 | 4.70E-10 |
| CELF4 | 0.08277 | 0.844883 | 3.351571 | 2.39E-28 | 4.06E-26 |
| CELF5 | 0.026476 | 0.104908 | 1.986379 | 8.55E-13 | 4.73E-12 |
| DDX17 | 81.13037 | 56.41145 | -0.52425 | 1.27E-10 | 5.43E-10 |
| DDX43 | 0.681298 | 0.40956 | -0.73421 | 5.92E-12 | 2.99E-11 |
| MRPL14 | 26.36111 | 48.24691 | 0.872025 | 6.65E-21 | 1.65E-19 |
| CPEB1 | 0.404774 | 0.622728 | 0.621483 | 0.001169 | 0.001882 |
| CPEB3 | 2.829257 | 1.830974 | -0.62781 | 7.23E-18 | 9.37E-17 |
| CPEB4 | 9.499983 | 5.629961 | -0.7548 | 1.15E-18 | 1.71E-17 |
| RPUSD3 | 3.226433 | 4.759386 | 0.560835 | 3.78E-21 | 1.03E-19 |
| RPL28 | 70.5359 | 105.152 | 0.576046 | 1.17E-18 | 1.73E-17 |
| DDX60 | 4.330073 | 6.18968 | 0.515473 | 5.39E-05 | 0.000103 |
| PABPC1L | 3.549623 | 5.332006 | 0.587013 | 4.75E-05 | 9.18E-05 |
| PABPC4L | 0.67906 | 1.155765 | 0.767237 | 9.95E-10 | 3.81E-09 |
| PABPC5 | 0.762259 | 0.493229 | -0.62802 | 1.73E-12 | 9.27E-12 |
| MEX3A | 0.313806 | 1.19714 | 1.931645 | 7.17E-23 | 3.05E-21 |
| MEX3B | 0.281876 | 0.398866 | 0.500844 | 5.14E-06 | 1.14E-05 |
| MEX3D | 3.993479 | 7.633151 | 0.934633 | 2.81E-18 | 3.90E-17 |
| PARP4 | 12.73026 | 23.86313 | 0.906521 | 7.69E-15 | 5.85E-14 |
| MRPS31 | 13.05972 | 9.136263 | -0.51545 | 5.70E-22 | 1.99E-20 |
| FBLL1 | 0.178067 | 0.864567 | 2.279561 | 3.44E-14 | 2.28E-13 |
| USB1 | 5.635306 | 8.874827 | 0.655225 | 1.83E-26 | 2.07E-24 |
| ISG20 | 4.863783 | 3.044738 | -0.67576 | 1.11E-05 | 2.33E-05 |
| AEN | 4.058018 | 6.126714 | 0.594338 | 1.16E-13 | 7.22E-13 |
| ARHGEF28 | 3.518257 | 1.489284 | -1.24024 | 6.81E-23 | 2.99E-21 |
| DZIP3 | 6.7025 | 4.025088 | -0.73568 | 3.82E-27 | 5.78E-25 |
| ZFR2 | 0.057844 | 0.189197 | 1.709648 | 8.44E-06 | 1.80E-05 |
| YBX3 | 11.69779 | 17.85602 | 0.610174 | 7.25E-12 | 3.62E-11 |
| ARL6IP4 | 0.723453 | 1.047076 | 0.533396 | 2.18E-06 | 5.04E-06 |
| YARS | 6.678071 | 10.69141 | 0.678948 | 9.93E-26 | 8.71E-24 |
| LRRFIP2 | 5.502267 | 3.804636 | -0.53227 | 1.74E-19 | 3.11E-18 |
| EIF4E1B | 0.000965 | 0.031706 | 5.037648 | 0.000278 | 0.000484 |
| PAPOLB | 0.017189 | 0.003726 | -2.20596 | 1.53E-20 | 3.53E-19 |
| RNASET2 | 10.18706 | 6.136841 | -0.73117 | 2.32E-19 | 3.90E-18 |
| NOLC1 | 27.49946 | 19.09489 | -0.52622 | 5.88E-21 | 1.48E-19 |
| NANOS1 | 2.963824 | 4.303626 | 0.538093 | 2.11E-05 | 4.26E-05 |
| NANOS3 | 0.478634 | 1.246814 | 1.381253 | 9.20E-19 | 1.42E-17 |
| HELZ | 3.787244 | 2.565811 | -0.56173 | 6.34E-20 | 1.23E-18 |
| MRPS25 | 9.199815 | 13.76411 | 0.581235 | 3.80E-16 | 3.77E-15 |
| IFIT2 | 3.105701 | 5.928863 | 0.932837 | 2.66E-09 | 9.26E-09 |
| IFIT5 | 5.718602 | 8.651611 | 0.597306 | 7.46E-09 | 2.45E-08 |
| IFIT3 | 6.54444 | 12.27024 | 0.906822 | 8.37E-09 | 2.71E-08 |
| NOVA2 | 0.554531 | 0.876428 | 0.660368 | 5.45E-07 | 1.35E-06 |
| SETX | 16.3098 | 10.84653 | -0.58851 | 4.13E-15 | 3.47E-14 |
| JAKMIP1 | 0.455452 | 0.226901 | -1.00523 | 2.18E-14 | 1.50E-13 |
