# Supplementary material for: Integrated analysis of RNA-binding proteins in thyroid cancer
Source: PLoS One. 2021 Mar 12;16(3):e0247836. doi: 10.1371/journal.pone.0247836 (PMC7954316; doi:10.1371/journal.pone.0247836)
Supplement: S2 Table — (DOCX) [file pone.0247836.s002.docx]

**S2 Table. GO and KEGG pathway analysis results.**

| **Up-regulated RBPs** | **P-value** | **Down-regulated RBPs** | **P-value** |
| --- | --- | --- | --- |
| regulation of cellular amide metabolic process | 1.24E-10 | regulation of translation | 2.03E-11 |
| RNA catabolic process | 6.69E-12 | regulation of cellular amide metabolic process | 1.26E-10 |
| RNA phosphodiester bond hydrolysis | 4.06E-11 | negative regulation of translation | 1.97E-10 |
| regulation of cellular amide metabolic process | 1.24E-10 | negative regulation of cellular amide metabolic process | 5.23E-10 |
| regulation of translation | 2.04E-10 | regulation of mRNA metabolic process | 1.55E-09 |
| defense response to virus | 2.88E-10 | 3'-UTR-mediated mRNA destabilization | 2.17E-09 |
| nucleic acid phosphodiester bond hydrolysis | 4.32E-09 | positive regulation of mRNA metabolic process | 1.20E-08 |
| regulation of mRNA catabolic process | 1.01E-08 | regulation of RNA splicing | 2.76E-08 |
| response to virus | 1.02E-08 | mRNA destabilization | 6.80E-08 |
| mRNA catabolic process | 3.44E-08 | RNA splicing | 9.41E-08 |
| P-body | 6.31E-08 | cytoplasmic ribonucleoprotein granule | 3.27E-09 |
| cytoplasmic stress granule | 0.002719 | ribonucleoprotein granule | 5.32E-09 |
| ribonucleoprotein granule | 9.18E-12 | P granule | 6.74E-09 |
| cytoplasmic ribonucleoprotein granule | 4.84E-12 | pole plasm | 6.74E-09 |
| P granule | 7.79E-05 | germ plasm | 6.74E-09 |
| pole plasm | 7.79E-05 | chromatoid body | 9.59E-08 |
| germ plasm | 7.79E-05 | cytoplasmic stress granule | 3.43E-06 |
| nucleolar part | 0.000106 | ribosome | 0.000378 |
| RNA polymerase II, core complex | 0.001774 | CCR4-NOT complex | 0.001587 |
| fibrillar center | 0.000238 | apical dendrite | 0.001587 |
| catalytic activity, acting on RNA | 6.02E-12 | helicase activity | 6.12E-10 |
| mRNA 3'-UTR binding | 2.53E-11 | mRNA 3'-UTR binding | 2.05E-09 |
| double-stranded RNA binding | 3.48E-09 | catalytic activity, acting on RNA | 2.19E-09 |
| translation regulator activity | 1.84E-08 | RNA helicase activity | 1.92E-08 |
| nuclease activity | 7.19E-08 | translation repressor activity, mRNA regulatory element binding | 2.64E-07 |
| ribonuclease activity | 9.69E-08 | nuclease activity | 1.15E-06 |
| mRNA 3'-UTR AU-rich region binding | 1.64E-07 | translation repressor activity | 2.80E-06 |
| AU-rich element binding | 2.43E-07 | mRNA 3'-UTR AU-rich region binding | 2.80E-06 |
| exonuclease activity | 0.007667 | translation regulator activity | 3.34E-06 |
| single-stranded RNA binding | 7.76E-06 | AU-rich element binding | 3.81E-06 |
| RNA transport | 3.08E-06 | mRNA surveillance pathway | 0.00039 |
| RNA degradation | 0.002768 |  |  |
| Influenza A | 0.003086 |  |  |
| mRNA surveillance pathway | 0.0048 |  |  |
| RNA polymerase | 0.005465 |  |  |
